# Supplementary material for: Causal Relationship between Aging and Anorexia Nervosa: A White-Matter-Microstructure-Mediated Mendelian Randomization Analysis
Source: Biomedicines. 2024 Aug 16;12(8):1874. doi: 10.3390/biomedicines12081874 (PMC11351342; doi:10.3390/biomedicines12081874)
Supplement: Supplementary file 1 [file biomedicines-12-01874-s001.zip › Supplementary Figure/Figure S4/Singe SNP plot of MR analysis for TL to ubm-b-2051.pdf]

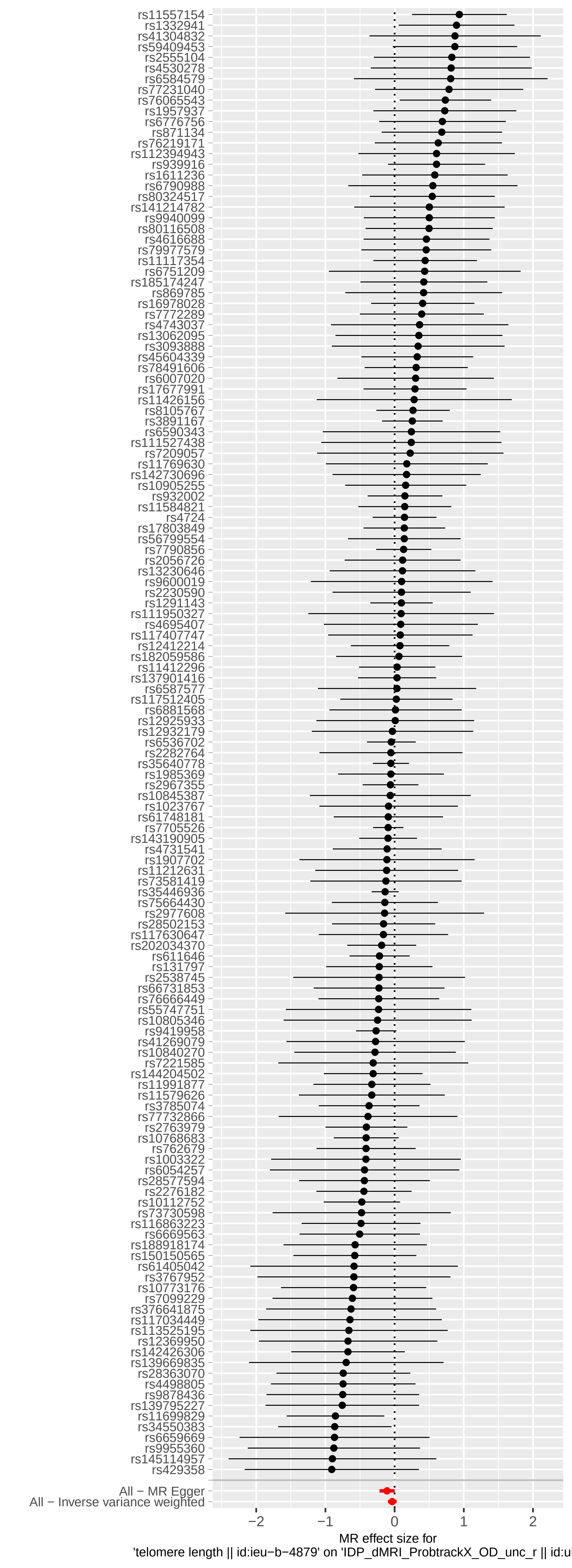

rs11557154
rs1332941
rs41304832
rs59409453
rs2555104
rs4530278
rs6584579
rs77231040
rs76065543
rs1957937
rs6776756
rs871134
rs76219171
rs112394943
rs939916
rs1611236
rs6790988
rs80324517
rs141214782
rs9940099
rs80116508
rs4616688
rs79977579
rs11117354
rs6751209
rs185174247
rs869785
rs16978028
rs7772289
rs4743037
rs13062095
rs3093888
rs45604339
rs78491606
rs6007020
rs17677991
rs11426156
rs8105767
rs3891167
rs6590343
rs111527438
rs7209057
rs11769630
rs142730696
rs10905255
rs932002
rs11584821
rs4724
rs17803849
rs56799554
rs7790856
rs2056726
rs13230646
rs9600019
rs2230590
rs1291143
rs111950327
rs4695407
rs117407747
rs12412214
rs182059586
rs11412296
rs137901416
rs6587577
rs117512405
rs6881568
rs12925933
rs12932179
rs6536702
rs2282764
rs35640778
rs1985369
rs2967355
rs10845387
rs1023767
rs61748181
rs7705526
rs143190905
rs4731541
rs1907702
rs11212631
rs73581419
rs35446936
rs75664430
rs2977608
rs28502153
rs117630647
rs202034370
rs611646
rs131797
rs2538745
rs66731853
rs76666449
rs55747751
rs10805346
rs9419958
rs41269079
rs10840270
rs7221585
rs144204502
rs11991877
rs11579626
rs3785074
rs77732866
rs2763979
rs10768683
rs762679
rs1003322
rs6054257
rs28577594
rs2276182
rs10112752
rs73730598
rs116863223
rs6669563
rs188918174
rs150150565
rs61405042
rs3767952
rs10773176
rs7099229
rs376641875
rs117034449
rs113525195
rs12369950
rs142426306
rs139669835
rs28363070
rs4498805
rs9878436
rs139795227
rs11699829
rs34550383
rs6659669
rs9955360
rs145114957
rs429358
All − MR Egger
All − Inverse variance weighted
−2
−1
0
1
2
MR effect size for
'telomere length || id:ieu−b−4879' on 'IDP_dMRI_ProbtrackX_OD_unc_r || id:u
